# Phosphorylation of Parkin at serine 65 is essential for its activation *in vivo*

**DOI:** 10.1098/rsob.180108

**Published:** 2018-11-07

**Authors:** Thomas G. McWilliams, Erica Barini, Risto Pohjolan-Pirhonen, Simon P. Brooks, François Singh, Sophie Burel, Kristin Balk, Atul Kumar, Lambert Montava-Garriga, Alan R. Prescott, Sidi Mohamed Hassoun, François Mouton-Liger, Graeme Ball, Rachel Hills, Axel Knebel, Ayse Ulusoy, Donato A. Di Monte, Jevgenia Tamjar, Odetta Antico, Kyle Fears, Laura Smith, Riccardo Brambilla, Eino Palin, Miko Valori, Johanna Eerola-Rautio, Pentti Tienari, Olga Corti, Stephen B. Dunnett, Ian G. Ganley, Anu Suomalainen, Miratul M. K. Muqit

**Affiliations:** 1 MRC Protein Phosphorylation and Ubiquitylation Unit, School of Life Sciences, University of Dundee, Dundee DD1 5EH, UK; 2 Dundee Imaging Facility, School of Life Sciences, University of Dundee, Dundee DD1 5EH, UK; 3 School of Medicine, University of Dundee, Dundee DD1 9SY, UK; 4 Research Programs Unit, Molecular Neurology, University of Helsinki, 00290 Helsinki, Finland; 5 Neuroscience Center, University of Helsinki, 00290 Helsinki, Finland; 6 Helsinki University Hospital, 00290 Helsinki, Finland; 7 The Brain Repair Group, Division of Neuroscience, School of Biosciences, Cardiff University, Wales CF10 3AX, UK; 8 CNRS, Inserm, Paris, France; 9 German Center for Neurodegenerative Diseases (DZNE), Bonn, Germany; 10 Neuroscience & Mental Health Institute, Neuroscience Division, School of Biosciences, Hadyn Ellis Building, Maindy Road, Cardiff CF24 4HQ, UK; 11 Department of Neurology, Helsinki University Hospital, Haartmaninkatu 4, Helsinki, FI 00290, Finland

**Keywords:** mitochondria, mitophagy, neurodegeneration, Parkin, Parkinson's disease, PINK1

## Abstract

Mutations in PINK1 and Parkin result in autosomal recessive Parkinson's disease (PD). Cell culture and *in vitro* studies have elaborated the PINK1-dependent regulation of Parkin and defined how this dyad orchestrates the elimination of damaged mitochondria via mitophagy. PINK1 phosphorylates ubiquitin at serine 65 (Ser65) and Parkin at an equivalent Ser65 residue located within its N-terminal ubiquitin-like domain, resulting in activation; however, the physiological significance of Parkin Ser65 phosphorylation *in vivo* in mammals remains unknown. To address this, we generated a *Parkin* Ser65Ala (S65A) knock-in mouse model. We observe endogenous Parkin Ser65 phosphorylation and activation in mature primary neurons following mitochondrial depolarization and reveal this is disrupted in *Parkin*^S65A/S65A^ neurons. Phenotypically, *Parkin*^S65A/S65A^ mice exhibit selective motor dysfunction in the absence of any overt neurodegeneration or alterations in nigrostriatal mitophagy. The clinical relevance of our findings is substantiated by the discovery of homozygous PARKIN (*PARK2*) p.S65N mutations in two unrelated patients with PD. Moreover, biochemical and structural analysis demonstrates that the Parkin^S65N/S65N^ mutant is pathogenic and cannot be activated by PINK1. Our findings highlight the central role of Parkin Ser65 phosphorylation in health and disease.

## 1. Introduction

Mutations in genes encoding PTEN-induced kinase 1 (PINK1) (human *PARK6*) and the ubiquitin E3 ligase Parkin (human *PARK2*) are causal for early onset Parkinson's disease (PD) with clinically indistinguishable phenotypes [1,2]. Patients typically exhibit a motor syndrome associated with nigrostriatal pathology that can occur in the absence or the presence of Lewy body inclusions [3]. Under specific conditions of mitotoxic stress in cultured cells, PINK1 and Parkin converge in a common signal transduction pathway to eliminate damaged mitochondria via autophagy, known as mitophagy [4–6]. Over the last decade, this pathway has been intensively studied to understand the mechanisms by which these enzymes regulate mitochondrial homeostasis [4–6]. In cell culture, PINK1 is activated upon mitochondrial depolarization that can be induced using mitochondrial uncoupling agents, e.g. antimycin A/oligomycin (A/O). This results in the recruitment and activation of Parkin at the outer mitochondrial membrane (OMM) [4–6]. PINK1 directly phosphorylates Parkin at a highly conserved serine 65 (Ser65) residue within its N-terminal ubiquitin-like domain (Ubl), as well as the equivalent Ser65 residue of ubiquitin (Ub). Phosphorylation of both Parkin and ubiquitin is required for maximal activation of Parkin E3 ligase activity [7–11]. Upon activation, Parkin ubiquitylates multiple substrates at the OMM leading to both *de novo* assembly and elongation of existing ubiquitin chains that are, in turn, phosphorylated by PINK1. Together, this generates a feed-forward enhancement of Parkin activation and mitochondrial ubiquitylation that heralds the recruitment of selective autophagy adaptors necessary for the completion of mitophagy [12–15].

Our present understanding of PINK1-dependent Parkin activation is largely predicated on *in vitro* observations and cell culture studies, which often exploit the over-expression of exogenous Parkin and/or PINK1 at supra-physiological levels [4–6]. These include the previous demonstration that Parkin Ser65 phosphorylation may not be essential for its complete E3 ligase activity [8,12] or its depolarization-induced mitochondrial translocation [7,10]. Despite a concerted body of work in this area from many laboratories, the physiological significance of Parkin Ser65 phosphorylation by PINK1 *in vivo* remains largely enigmatic. Furthermore, additional substrates for PINK1 have been widely reported [16] but, to date, it is unknown whether inactivation of PINK1-dependent substrate phosphorylation is sufficient to recapitulate neurodegeneration *in vivo*. Models of germ-line *Parkin* knockout mice only exhibit mild phenotypes, with chronic mitotoxicity or extreme stress required to elicit PD-relevant neurological phenotypes [17–21]. To test the hypothesis that Parkin Ser65 phosphorylation is critical for its activation *in vivo*, we generated a *Park2* Ser65Ala knock-in mouse (*Parkin*^S65A/S65A^) and performed an extensive multi-parametric characterization of this model. We demonstrate that phosphorylation of Ser65 is critical for endogenous Parkin activation and Ser65-phosphorylated ubiquitin (hereafter referred to as phospho-ubiquitin) accumulation in primary cells, including neurons. Loss of endogenous Parkin Ser65 phosphorylation and activity results in selective locomotor impairments, accompanied by a mild striatal-specific mitochondrial defect *in vivo*. Furthermore, crossing our *Parkin*^S65A/S65A^ mice with the recently described *mito*-QC mitophagy reporter model [22] revealed that basal mitophagy in nigrostriatal dopamine neurons proceeds normally in *Parkin*^S65A/S65A^ mice, suggesting that the regulation of basal mitophagy is unaffected by the loss of Parkin E3 ligase activity in dopaminergic neurons. The pathophysiological significance of Parkin Ser65 phosphorylation in mammals is ultimately substantiated by the clinical discovery of two unrelated patients with relatively early onset PD harbouring a homozygous *PARK2* Ser65Asn (Parkin^S65N^) mutation. Characterization of patient-derived primary cells demonstrates that the Parkin S65N mutation is inactive, suggesting that the loss of PINK1-dependent Parkin Ser65 phosphorylation and subsequent inactivation in humans is sufficient to cause PD. Taken together, our data demonstrate that the phosphorylation state of Parkin at Ser65 is crucial for mitochondrial integrity and human nigrostriatal function.

## 2. Results

### 2.1. Endogenous Parkin activation is abolished in *Parkin*^S65A/S65A^ knock-in mice

**Figure 1.**
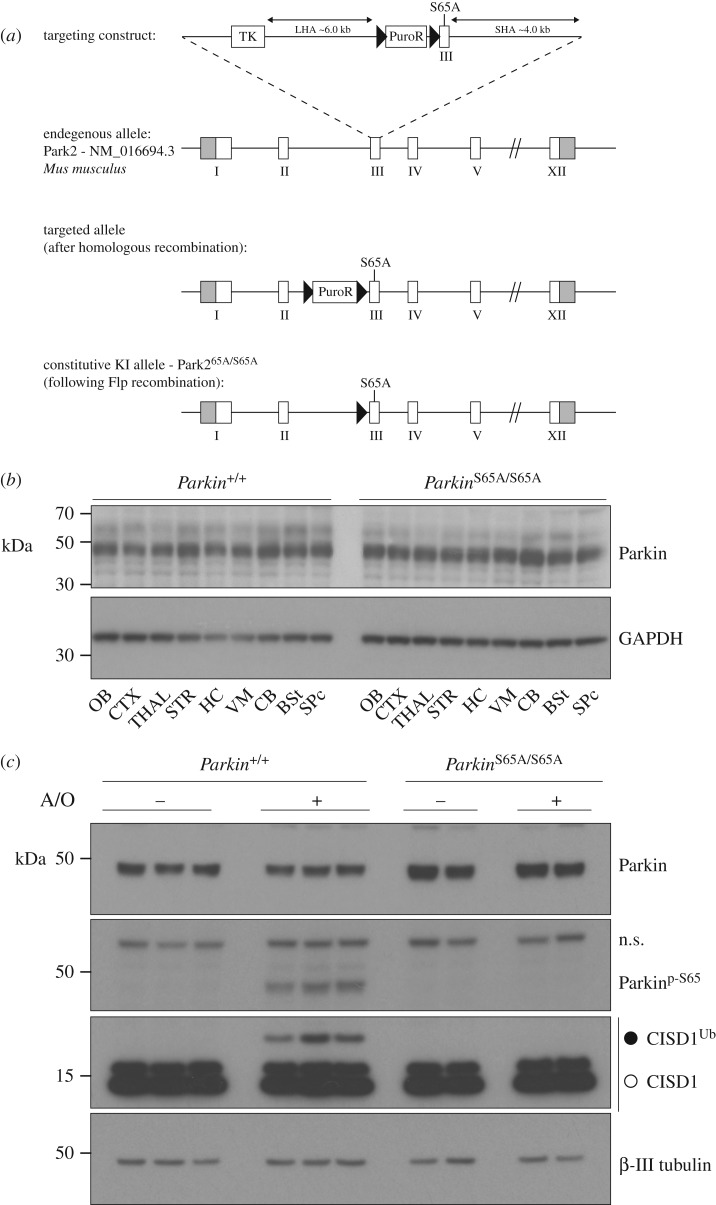
Generation and validation of the *Parkin*^S65A/S65A^ mouse. (*a*) Depiction of targeting strategy to generate a constitutive knock-in of Ser65Ala (S65A) point mutation in the *Mus musculus Park2* (Parkin) gene (targeting strategy based on NCBI transcript NM_016694.3). Exon 1 contains the translation initiation codon. The S65A mutation was introduced into exon 3. The *Park2*^S65A/S65A^ knock-in (KI) allele was generated following Flp-mediated recombination. (*b*) Rostro-caudal expression analysis of Parkin protein expression in the mouse central nervous system (CNS) under basal conditions. Immunoblot analysis of sub-dissected CNS regions from adult wild-type and *Parkin*^S65A/S65A^ mice. OB, olfactory bulb; CTX, neocortex; THAL, thalamus; STR, striatum; HC, hippocampus; VM, ventral midbrain; CB, cerebellum; BSt, brainstem; SPc, spinal cord. (*c*) Parkin Ser65 phosphorylation is essential for Parkin activation in primary neurons. Mature (21 DIV) primary cortical neuron cultures were established from wild-type and *Parkin*^S65A/S65A^ mice, and stimulated for 3 h with a combination of antimycin A (10 µM)/oligomycin (1 µM). Whole-cell extracts were subjected to SDS-PAGE and immunoblot analysis with anti-Parkin, anti-phospho-Ser65 Parkin, anti-CISD1 and anti-β-III tubulin antibodies.

To test the hypothesis that Parkin Ser65 phosphorylation is central to Parkin activation and neuronal homeostasis *in vivo*, we generated a knock-in mouse model in which the codon encoding Parkin Ser65 was altered by homologous recombination to alanine to prevent Parkin protein phosphorylation by PINK1 (figure 1*a*). Homozygous *Parkin*^S65A/S65A^ mice were born at expected Mendelian frequencies and wild-type and mutant mice were indistinguishable in terms of gross measures of growth, weight and development (data not shown).

**Figure 2.**
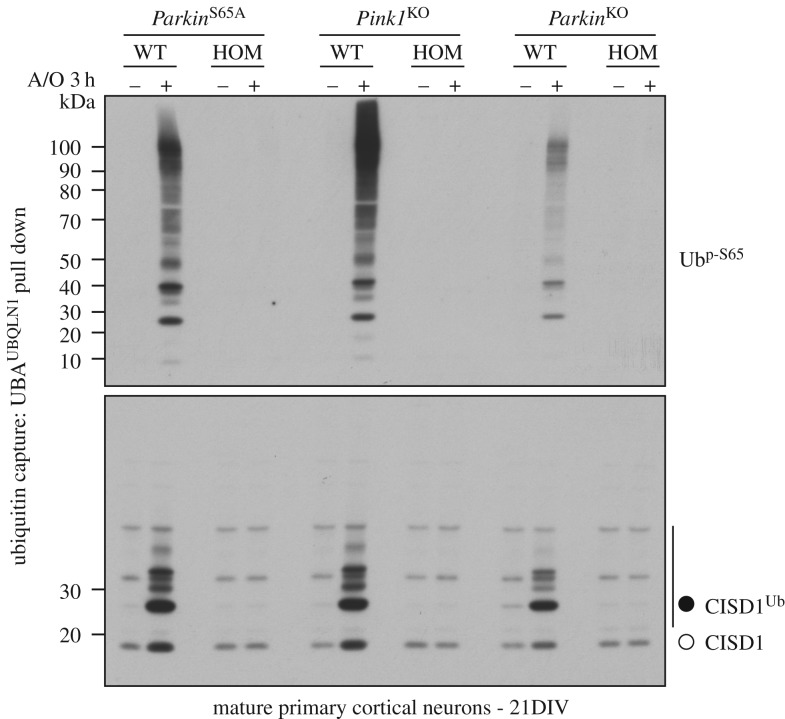
Phospho-ubiquitin levels are diminished in *Parkin*^S65A/S65A^ mature primary neurons. Immunoblots showing comparative analysis of phospho-Ser65 ubiquitin levels in mature (21 DIV) primary cortical neuron cultures from the following groups: *Parkin*^S65A/S65A^ mice and matched wild-type controls; *Pink1* knockout (KO) and wild-type mice and *Parkin* knockout and wild-type mice. Cultures were stimulated with A/O for 3 h before lysis and membrane enrichment. Protein extracts were enriched for ubiquitylated substrates by incubating with ubiquitin-binding resin derived from His-Halo-Ubiquilin UBA-domain tetramer (HALO-UBA^UBQLN1^). Enriched lysates were subjected to immunoblotting with anti-phospho-Ser65 ubiquitin and anti-CISD1 antibodies.

The expression and stability of the endogenous mutant Parkin^S65A^ protein in *Parkin*^S65A/S65A^ mice was comparable to wild-type mice across all brain regions (olfactory bulb, cortex, thalamus, striatum, hippocampus, ventral midbrain, cerebellum, brainstem and spinal cord) and extra-neural tissues (heart, spleen, lung and pancreas) (figure 1*b* and electronic supplementary material, figure S1). To assess how the S65A mutation affects Parkin ubiquitin E3 ligase activity, we undertook biochemical analysis of endogenous substrate ubiquitylation in cultured primary neurons. Given the availability of reliable reagents and building upon our previous work in this area [11,23], we focused our attention on the mitochondrial Fe/S domain-containing protein CISD1, a well-described Parkin substrate, in addition to monitoring endogenous PINK1-mediated Parkin Ser65 phosphorylation. We have previously observed that endogenous Parkin is expressed at low levels in mouse embryonic fibroblasts (MEFs) which limits the robust detection of endogenous Parkin signalling [23]. However, in primary cortical neuron cultures established from E16.5 mouse embryos cultured to maturity for 21 days *in vitro* (DIV), endogenous Parkin phosphorylation and substrate ubiquitylation are reliably detectable after combined antimycin A and oligomycin stimulation (to trigger mitochondrial depolarization; hereafter referred to as A/O) [24]. Upon treatment of mature (21 DIV) cortical neurons with A/O for three hours (3 h), we observed complete loss of CISD1 ubiquitylation in *Parkin*^S65A/S65A^ neurons compared to wild-type, as measured by anti-CISD1 immunoblotting (figure 1*c*). Immunoblot analysis using anti-phospho-Ser65 Parkin antibodies confirmed the loss of Parkin phosphorylation at Ser65 in mutant neurons (figure 1*c*). We also assessed endogenous ubiquitylation status of additional Parkin substrates including Mitofusin2, Miro2 and VDAC1; however, we were unable to detect robust signal in neurons in whole-cell lysates or following HALO-Ubiquilin1 UBA-domain tetramer (UBA^UBQLN1^) TUBE-pulldown experiments (electronic supplementary material, figure S2). This may be due to their low stoichiometry of ubiquitylation, demonstrated recently in human neurons by quantitative mass spectrometry [25]. We next asked whether loss of Parkin activity in *Parkin*^S65A/S65A^ neurons influenced the PINK1-dependent phosphorylation of ubiquitin at Ser65 and its subsequent accumulation. We undertook comparative biochemical analyses of mature primary neuron cultures established from homozygous *Parkin*^S65A/S65A^, *Pink1* knockout and *Parkin* knockout mice with their respective corresponding wild-type littermate controls. Consistent with previous studies in proliferating cell lines, we observed complete loss of Ser65-phosphorylated ubiquitin (phospho-ubiquitin) and CISD1 ubiquitylation in *Pink1* knockout neurons, as judged by immunoblotting of HALO-UBA^UBQLN1^ pulldowns with anti-phospho-Ser65 ubiquitin and anti-CISD1 antibodies (figure 2). Strikingly, we did not observe phospho-ubiquitin accumulation in either *Parkin*^S65A/S65A^ or *Parkin* knockout neurons that were associated with loss of CISD1 substrate ubiquitylation (figure 2). These data support the Parkin-dependent feed-forward model of phospho-ubiquitin accumulation [12,15]. Similar findings were recently reported in CRISPR/Cas9-mediated S65A mutant human embryonic stem (ES) cell-derived neurons stimulated with A/O in which phospho-ubiquitin was not detectable by immunoblotting, although the authors were able to detect phospho-ubiquitin by mass spectrometry [25]. To confirm this finding in other primary mouse cell types, we exploited a tractable culture paradigm of mouse primary adult lung fibroblast cultures [26]. We stimulated adult primary fibroblast cultures from *Parkin*^S65A/S65A^ and *Pink1* knockout mice with the protonophore carbonyl cyanide m-chlorophenyl hydrazone (CCCP) for 18 h, which dissipates mitochondrial membrane potential similar to A/O. As Parkin protein levels were lower in primary lung fibroblasts compared with neurons, we performed an additional mitochondrial enrichment step prior to ubiquitin capture by HALO-UBA^UBQLN1^ pulldown. Consistent with our results in neurons, we did not observe the accumulation of phospho-ubiquitin or CISD1 ubiquitylation in either *Parkin*^S65A/S65A^ or *Pink1* knockout fibroblasts compared with wild-type cultures (electronic supplementary material, figure S3). Overall, our data indicate that phosphorylation of Parkin Ser65 by PINK1 is essential for both activation of its E3 ligase activity and the robust generation of phospho-ubiquitin in mature mammalian neurons.

### 2.2. *Parkin*^S65A/S65A^ knock-in mice exhibit selective motor impairments

**Figure 3.**
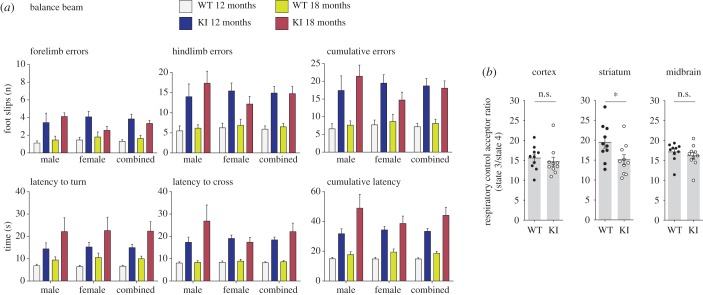
*Parkin*^S65A/S65A^ mice exhibit selective impairments on a sensitive task of voluntary motor function. (*a*) Balance beam performance in *Parkin*^S65A/S65A^ (KI) and wild-type mice at 12 months and 18 months of age. Animals were assessed by their ability to reach a platform by competently traversing a raised and tapered beam. The performance was recorded by the number of errors scored as slips (forelimb, hindlimb and combined). *Parkin*^S65A/S65A^ mice made significantly more forelimb errors and hindlimb errors at 12 and 18 months (*p* < 0.01) than their WT littermates. The *Parkin*^S65A/S65A^ mice also demonstrated an age-related decline in their ability to orientate themselves on the beam end and cross the beam, resulting in longer latencies on both measures compared to WT littermates (*p* < 0.01). Error bars represent the standard error of the mean. (*b*) Striatal RCR is affected in *Parkin*^S65A/S65A^ mice. Mitochondrial respiratory acceptor control ratios in the striatum, midbrain, and cortex of 1-year-old aged wild-type and *Parkin*^S65A/S65A^ mice (*n* = 10 per group).

Since our *Parkin*^S65A/S65A^ mice have been engineered with an inactivating point mutation in the endogenous *Park2* locus and express comparable levels of Parkin protein, we speculated that our model may bypass epistatic compensation induced by the loss of total protein in traditional knockout models. We therefore undertook a comprehensive behavioural characterization of our *Parkin*^S65A/S65A^ mice to investigate if the loss of endogenous Parkin phosphorylation could influence striatal function *in vivo*. Overall, *Parkin*^S65A/S65A^ mice exhibited normal gross development and ageing in terms of neurological and behavioural function. We employed the raised balance beam as a more sensitive and powerful test of voluntary locomotor function, balance and coordinated limb use in our mice. Strikingly, we observed that *Parkin*^S65A/S65A^ mice at 12 and 18 months of age exhibited consistent impairments in all measures of balance beam performance compared with wild-type control mice (figure 3*a*). Both the latency to turn towards the goal box when placed on the beam end (AGE × GENO: *F*_1,32_ = 11.41, *p* < 0.01), and the subsequent latency to cross the beam (AGE × GENO × SEX: *F*_1,32_ = 12.82, *p* < 0.01) were slower for *Parkin*^S65A/S65A^ mice compared with wild-type controls, with mutant males being slower to traverse the beam than their female counterparts (figure 3*a*). Furthermore, quantitation of foot-slips while traversing the balance beam demonstrated that the number of forelimb (GENO: *F*_1,47_ = 29.08, *p* < 0.01) or hindlimb (GENO: *F*_1,47_ = 38.52, *p* < 0.01) slips made by *Parkin*^S65A/S65A^ mice was greater than controls (figure 3*a*), but this phenotype was not exacerbated at 18 months. In addition to deficits in motor function, the impairments observed in *Parkin*^S65A/S65A^ mice to complete the task, particularly during the ‘beam turn’ aspect, could also indicate an underlying, yet subtle defect in cognitive planning (figure 3*a*). Rotarod testing at 12 and 18 months of age did not reveal any substantial differences between *Parkin*^S65A/S65A^ and wild-type mice (AGE × GENO: *p* = 0.058) (electronic supplementary material, figure S4). Additionally, *Parkin* mutant mice did not exhibit any impairments in gait in comparison to wild-type mice in terms of stride length and stride width (electronic supplementary material, figure S4). Although the rotarod is routinely used to assess locomotor function in rodents, its lack of sensitivity and confounding variables are well recognized [27]. Overall, these data demonstrate that endogenous Parkin inactivation *in vivo* results in selective deficits in a sensitive test of voluntary motor function. Consistent with our results, deficits in fine motor coordination were reported in one previous characterization of *Parkin* knockout mice generated by targeted deletion of exon 3 [17]; however, in another independently generated exon 3 deleted knockout model, no motor deficits were reported although these mice exhibited learning and memory deficits [28]. More recently, similar fine motor deficits were found in *Pink1* knockout mice in the absence of nigrostriatal degenerative pathology [29], further underscoring the importance of performing more sensitive behavioural testing in mouse models of PD. In our study, we were unable to determine the age-of-onset of the motor deficits, although it was reported that *Pink1* knockout mice take approximately six months to exhibit detectable locomotor dysfunction [29].

Given the mitochondrial-centric functions ascribed to the PINK1–Parkin dyad, we next investigated if the selective motor phenotype in our *Parkin*^S65A/S65A^ mice could be attributed to mitochondrial dysfunction. We assayed mitochondrial respiration in discrete adult brain regions from 3-month old and 12-month-old wild-type and mutant mice using high-resolution (Oroboros Oxygraph-2k) respirometry [20]. In 3-month-old mice, we observed no difference in the respiratory control ratio (RCR) defined as State3_ADP_/State4 that reflects mitochondrial oxidative phosphorylation coupling efficiency independently of mitochondrial content [30,31] (electronic supplementary material, figure S5*a*). By contrast, we detected a highly selective and consistent difference in mitochondrial RCR between the striatum of 12-month-old *Parkin*^S65A/S65A^ and wild-type mice but not in other brain regions including cortex (figure 3*b* and electronic supplementary material, figure S5*b*). This is consistent with previous reports of impaired mitochondrial respiration in select brain regions of aged *Parkin* knockout mice [20], and suggests that Parkin Ser65 phosphorylation contributes to mitochondrial integrity in this brain region.

### 2.3. Nigrostriatal integrity and basal mitophagy levels are normal in *Parkin*^S65A/S65A^ knock-in mice

**Figure 4.**
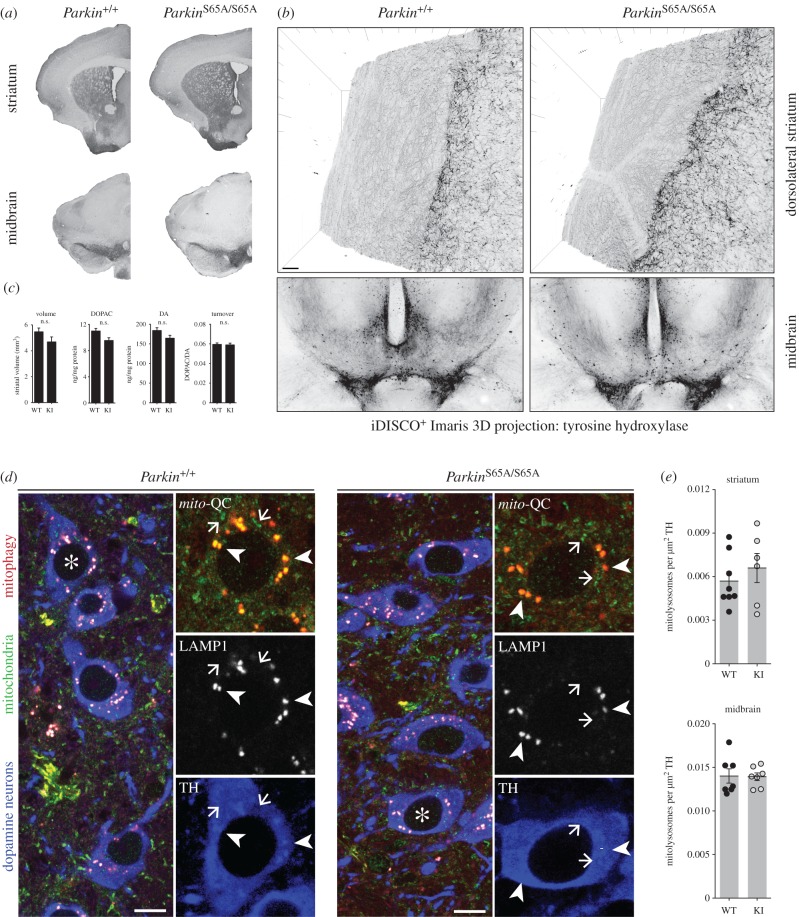
*Parkin*^S65A/S65A^ mice do not exhibit nigrostriatal degeneration or defective mitophagy. (*a–c*) Striatal innervation is indistinguishable between wild-type and *Parkin*^S65A/S65A^ (KI) mice. (*a*) No differences in neuron number or striatal innervation were observed by anti-tyrosine hydroxylase (TH)-based immunohistochemistry analysis of midbrain and striatum between genotypes. (*b*) Striatal innervation analysis of TH-positive DA neurons within intact brains processed by iDISCO^+^. Arborization of DA neurons is indistinguishable between wild-type and KI mice. (*c*) No differences were observed in striatal volume between genotypes. Error bars represent standard errors of the mean. Striatal dopamine levels are unaltered in *Parkin*^S65A/S65A^ mice. HPLC analysis revealed no differences in dopamine (DA) and 3,4-dihydroxyphenylacetic acid (3,4-DOPAC) levels between wild-type and KI mice (*n* = 10 per genotype). Data represent mean values ±s.e.m. n.s., not significant. (*d*) Basal mitophagy is unaltered by endogenous Parkin activation *in vivo*. Maximum *z*-projections of midbrain DA neurons from *mito*-QC wild-type and KI mice immunolabelled with antibodies to TH (blue) and LAMP1 (greyscale). Arrows point to mitochondria and arrowheads point to mitochondria associated with (autophago)lysosomes/mitolysosomes as defined by co-localization of LAMP1 and *mito*-QC staining. Mitochondrial delivery to lysosomes (mitophagy) is associated with quenching of GFP signal of the *mito*-QC reporter and residual mCherry only signal intensity. Scale bar 5 µm. Asterisks indicates cells within insets. (*e*) Quantitation revealed no differences between mitophagy in DA cell bodies or axons. Results are expressed as mean values ± s.e.m.

The observed locomotor and striatal mitochondrial dysfunction in *Parkin*^S65A/S65A^ mice suggested that endogenous Parkin inactivation could also negatively impact nigrostriatal integrity *in vivo*. We next performed histological analysis of the substantia nigra of aged (approx. 18 months) *Parkin*^S65A/S65A^ and wild-type mice to investigate if the impaired viability of nigrostriatal dopaminergic (DA) neurons might account for the selective motor defects in *Parkin*^S65A/S65A^ mice. Inactivation of endogenous Parkin did not influence striatal anatomy or volume in aged mice (figure 4*a–c*). Nigrostriatal projections are highly complex, with a single DA neuron capable of innervating up to 2% of its terminal target field [5,32]. Although total DA neuron numbers were comparable between *Parkin*^S65A/S65A^ mice and controls (data not shown), it is conceivable that differences in target-field arborization could account for the observed motor defect in our mutant animals. At a gross neuroanatomical level, conventional chromogenic tyrosine hydroxlase (TH)-immunohistochemistry revealed indistinguishable striatal innervation between genotypes (figure 4*a*); however, this approach does not permit a high-resolution analysis of the nigrostriatal projection. To determine whether *Parkin* inactivation could impact the ontogeny and arborization of DA neurons *in vivo*, we employed whole volume imaging of the entire nigrostriatal pathway within intact, optically cleared adult brains using iDISCO^+^ [22,33,34]. This approach enabled utilization of confocal microscopy to assess the degree of innervation and overall connectivity between aged *Parkin*^S65A/S65A^ mice and littermate control mice at high resolution. Using this approach, we successfully resolved DA projections in wild-type and *Parkin*^S65A/S65A^ mice; however, we observed no differences between groups (figure 4*b*). Dysfunction in non-neuronal cells is also a reported feature of PD mouse models [35]. However, immunohistochemical labelling of microglia and astroglia by Iba1 and GFAP, respectively, revealed no overt differences between wild-type and *Parkin*^S65A/S65A^ mice (data not shown). To further investigate nigrostriatal function, we next profiled catecholamine neurotransmission in our mutant mice. We rapidly dissected striatal tissue from age-matched wild-type and *Parkin^S65A/S65A^* littermates and subjected these to HPLC analysis. We observed comparable levels of DA and DA turnover in wild-type and *Parkin*^S65A/S65A^ striata (figure 4*c*), indicating that in our mouse model, genetic ablation of endogenous *Parkin* phosphorylation and activation is not sufficient to impact DA neurotransmission *in vivo*. Collectively, these data demonstrate that endogenous Parkin inactivation results in a selective locomotor defect in the absence of structural nigrostriatal dysfunction *in vivo*. Importantly, our findings highlight the importance of Parkin activation under steady-state conditions *in vivo*.

Parkin has received most attention for its role in modulating mitochondrial quality control [5]. Maximal activation of Parkin E3 ligase activity leads to ubiquitylation of myriad substrates on the mitochondrial outer membrane, that engage autophagy receptors and drive the elimination of damaged mitochondria via mitophagy [4–6]. This widely accepted and reproducible model is largely based on *in vitro* findings, however recent advances in mouse genetics using the *mito-*QC and mt-Keima reporter models have revealed the physiological nature of basal mitophagy *in vivo* [22,36,37]. Furthermore, DA neurons have recently been shown to undergo high levels of mitophagy *in vivo* which occurs independently of PINK1 [38,39]. To investigate the regulation of basal mitophagy in the context of endogenous *Parkin* inactivation in mammals, we crossed *Parkin*^S65A/S65A^ mice with the *mito*-QC mitophagy reporter model to generate *Parkin*^S65A/S65A^-*mito*-QC mice. An assessment of wild-type and S65A *mito*-QC animals revealed that basal mitophagy in nigrostriatal DA cell bodies and projections was indistinguishable between wild-type and mutant mice (figure 4*d,e*). These data reveal that nigrostriatal mitophagy is unaffected in a mouse model of endogenous *Parkin* inactivation exhibiting selective defects in motor and mitochondrial function.

### 2.4. *PARK2*^S65N^ mutations cause human Parkinson's disease via direct disruption of the PINK1–Parkin axis

Human patients with *PARK2* mutations typically present with early onset PD displaying slow progression and sustained response to L-DOPA. Mutations in several key regulatory residues of Parkin have previously been identified, including Cys431Phe which disrupts the catalytic acceptor cysteine within the RING2 domain [40]. However, to date, no mutations affecting the Ser65 phosphorylation site have been reported. Here we report the first clinical and genetic evidence of PD associated with homozygous mutations at p.S65N, the critical node of the PINK1–Parkin pathway.

### 2.5. Case series

#### 2.5.1. Case 1

**Figure 5.**
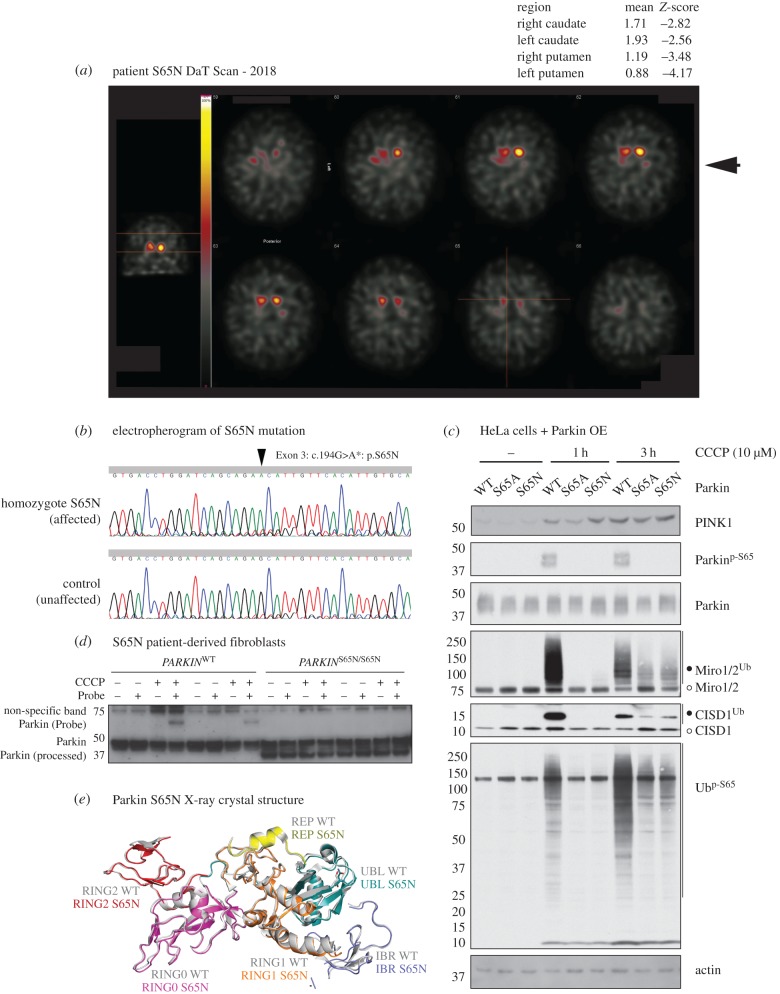
Discovery and characterization of case 1: a human PD-causing Parkin mutation at Ser65. (*a*) Dopamine transporter imaging (DaT scan) results with region-based semi-quantitative analysis. ^123^I-FP-CIT brain single-photon emission computed tomography (SPECT) or DaT scan imaging measures DAT density in presynaptic terminals of the DA neurons projecting from the substantia nigra to dorsal striatum (caudate nucleus and putamen) thereby enabling assessment of the structural integrity of the nigrostriatal pathway in humans. DaT SPECT imaging was performed three hours after an intravenous injection of 186 MBq ^123^I-FP-CIT using a double head gamma camera with a fan beam collimator. Thyroidal uptake was blocked by 300 mg of oral potassium perchlorate before the tracer injection. Occipital lobe was used as a reference region of non-specific binding. The *Z*-scores in the top right of the figure indicate significant deviation from the population mean values for both the left and right caudate and putamen. In the putamen, the DAT density was slightly more reduced, which is typical of the rostro-caudal pattern of uptake loss in PD. (*b*) Electropherogram detailing S65N mutation data (Exon 3 G > A). (*c*) Parkin S65A and S65N mutants display reduced Parkin activation in response to PINK1 activation. Flp-In T-Rex-HeLa cells stably expressing wild-type (WT), S65A and S65N Parkin were induced with 0.1 µg ml^–1^ doxycycline for 24 h prior to stimulation with DMSO or 10 µM CCCP at indicated timepoints (1 h or 3 h). Whole-cell lysates were subjected to immunoblot analysis using the indicated antibodies. For detection of ubiquitylation (marked by open circles), lysates were subjected to ubiquitylated-protein capture by His-Halo-Ubiquilin1 UBA-domain tetramer (HALO-UBA^UBQLN1^), prior to immunoblot with anti-CISD1 (Proteintech Europe) and anti-Miro1/2 (DSTT, S531D, 5th bleed). (*d*) Activity-based profiling of Parkinson's disease associated Parkin S65N patient-derived fibroblasts. Primary fibroblasts derived from skin biopsies from a healthy subject (WT) and a PD patient harbouring a Parkin S65N homozygous mutation were profiled with E2-ubiquitin-based probe to monitor Parkin E3 transthiolation activity as described previously [41]. Mitochondrial depolarization does not activate the PINK1–Parkin pathway in Parkin S65N patient cells (CCCP treatment (3 h, 10 µM)). (*e*) Composite schematic illustrating the X-ray crystal structure of the Parkin S65N disease mutant, superimposed over the wild-type structure (PDB ID: 5C1Z, Grey). ParkinS65N structure is depicted in colour: UBL (teal), RING0 (magenta), RING1 (orange), IBR (blue), REP (yellow) and RING2/Rcat (red).

We report a 71-year-old Finnish male, diagnosed with early onset PD at the age of 40 years with no reported family history of Parkinson's. His symptomatic presentation included rigidity of the lower limbs that was initially more marked on the left-hand side, and he exhibited shortness of steps. He has continued to experience rigidity throughout his disease course, without the development of tremor. Recently, the patient has suffered from a mild gait disturbance with occasional freezing and postural instability*.* Overall, the progression of his illness has been exceptionally slow with a sustained response to medication that currently comprises: levodopa (300–400 mg d^−1^), pramipexole (2.1 mg d^−1^) and selegiline (10 mg d^−1^). On examination during the years 2016–2017, he was categorized at stage 2.5 of the modified Hoehn and Yahr Scale, with a United Parkinson's Disease Rating Scale (UPDRS) score of 41/199 (electronic supplementary material, table S1) Dopamine transporter (DaT) single-photon emission computerized tomography (SPECT) of the brain demonstrated reduced density of DA synaptic terminals in the caudate and putamen consistent with degenerative PD (figure 5*a*).

Targeted next-generation sequencing (NGS) revealed that the patient carried a novel homozygous c.194G > A variant in exon 3 of the *PARK2* gene causing an amino acid change p.Ser65Asn (S65N) in the PARKIN protein (figure 5*b*). The variant is very rare: only two heterozygous carriers among 122 271 subjects were found in the Genome Aggregation Database (gnomAD) with an allelic frequency of 8.2 × 10^−6^*.* In the Exome Aggregation Consortium (ExAC) database, two heterozygotes were found among 60 691 subjects (allele frequency 1.6 × 10^−5^). Furthermore, the variant site is highly conserved in vertebrates (electronic supplementary material, figure S6*a*), and in *silico* analysis of the variant by the method of Combined Annotation Dependent Depletion (CADD) [42] predicted that the mutation is deleterious with a CADD C-score of 25.5. We analysed coding variants in 82 selected PD-associated loci (electronic supplementary material, table S2) with a CADD C-score of greater than 20 and population carrier frequency less than 1%, but did not find any other likely pathogenic variant in S65N-M70.

#### 2.5.2. Case 2

A 60 year-old Caucasian female diagnosed with PD at the age of 54 was identified from the Parkinson's Progression Markers Initiative (PPMI). Her initial clinical features were bradykinesia and gait difficulty on the right side. She exhibited characteristic but mild motor symptoms for PD and no atypical features have been observed. Twelve months following her diagnosis, she was commenced on pramipexole resulting in a positive and sustained response (current dosing 2.25 mg of pramipexole/day). At her latest examination, the patient was categorized at Stage 2 of the Hoehn and Yahr Scale, and she has also been examined using the Unified Parkinson's Disease Rating Scale (MDS-UPDRS) for 5 years (scores from latest examination are provided in electronic supplementary material, table S1).

The patient has also been followed up by DaT imaging for four years, which has demonstrated reduced density of DA synaptic terminals in the caudate and putamen consistent with DA denervation (striatal binding ratio (SBR) calculations from the latest session are provided in electronic supplementary material, table S1).

Genetic analysis of the individual revealed a homozygous Parkin p.S65N mutation. Analysis of 82 PD-associated loci revealed three other gene variants (in *POLG*, *MC1R* and Glucocerebrosidase (*GBA*) (electronic supplementary material, table S3) that passed our filtering criteria (CADD C-score greater than 20, carrier frequency less than 1%). A heterozygous recessive variant (p.G268A) in *POLG* has been linked to autosomal recessive/sporadic progressive external ophthalmoplegia (PEO) in compound heterozygous or homozygous form [43,44], but also described as single-heterozygous in a child with a syndrome including Parkinsonism born from consanguineous parents (probable autosomal recessive mode of inheritance) [45]. She also carried another *POLG* variant, a rare inframe deletion (exon2:c.153_158del:p.Gln54_Gln55del) in a tandem repeat region of *POLG*; however, this is likely a benign variant, given a CADD C-score of 6.2. Overall, we did not consider the heterozygous p.G268A *POLG* mutation as disease-causing in the patient. A rare heterozygous variant (p.R142H) was detected in the *MC1R* gene. This gene was initially found to be associated with PD, but subsequent studies with large patient cohorts have not supported this finding [46–49]. Furthermore, the p.R142H variant has to date only been associated with red hair and not PD [46]. Thus, we did not consider p.R142H being a disease-causing variant in this patient. By contrast, a heterozygous p.N409S variant in *GBA* was detected, which is a recognized susceptibility factor for PD [50] although the variant does not always lead to PD [51]. Therefore, we conclude that the PARKIN p.S65N mutation is likely to be the major disease-causing variant in the patient, although we cannot exclude that the GBA p.N409S variant may contribute to pre-disposition and/or the clinical phenotype of the patient.

#### 2.5.3. Functional analysis of the PARKIN^S65N/S65N^ mutation

We initially assessed the pathogenicity of the Parkin^S65N/S65N^ mutation by assaying PINK1–Parkin signalling in Flp-In TRex HeLa cells, engineered with stable expression of either wild-type Parkin, a non-phosphorylatable S65A mutant and the S65N mutant of Parkin. Following the induction of Parkin expression, cells were treated with 10 µM CCCP or DMSO for 1 or 3 h. Immunoblotting of protein extracts demonstrated that the S65N mutation was functionally equivalent to the S65A mutation, leading to both a loss of both Parkin activation (as judged by reduced CISD1 and Miro1/2 substrate ubiquitylation) and reduced phospho-ubiquitin accumulation (figure 5*c*). We next interrogated the influence of the S65N mutation on endogenous Parkin function, by assaying its activity and downstream signalling in patient-derived cells upon mitochondrial depolarization. Primary fibroblast cultures were established from a skin biopsy of the Finnish patient with homozygous Parkin S65N mutation, and an unaffected human control. Cultures were treated with 10 µM CCCP or DMSO for 3 h to induce PINK1 stabilization and activation (electronic supplementary material, figure S6*b*). Immunoblotting with anti-phospho-Ser65 Parkin antibodies demonstrated that Parkin phosphorylation was abolished in cells expressing S65N (electronic supplementary material, figure S6*b*). This was associated with substantial reduction of Parkin E3 ligase activity as judged directly by ubiquitylation of CISD1 in *PARKIN*^S65N/S65N^ fibroblasts (electronic supplementary material, figure S6*b*). Furthermore, we observed reduced accumulation of phospho-ubiquitin in patient fibroblasts compared with control cells, indicating reduced Parkin E3 ligase activity (electronic supplementary material, figure S6*b*). We further confirmed loss of Parkin E3 activity in patient-derived primary cultures by deploying a chemical probe that measures transthiolation activity of Parkin via covalent labelling of its active site cysteine residue Cys431 [41]. The probe revealed robust labelling of activated wild-type human Parkin in control fibroblasts, whereas this was completely disrupted in *PARKIN*^S65N/S65N^ mutant fibroblasts (figure 5*d*). Interestingly upon mitochondrial depolarization, we observed a partial reduction in mitochondrial PINK1 stabilization in the *PARKIN*^S65N/S65N^ mutant fibroblasts (electronic supplementary material, figure S7) and this is consistent with similar findings observed in CRISPR/Cas9 mediated S65A mutant human ES cell-derived neurons stimulated with A/O [25].

We next investigated the effect of the endogenous *PARKIN*^S65N/S65N^ mutation on basal and depolarization-induced mitophagy. We first generated a stable mitophagy reporter cell line via retroviral transduction of *mito*-QC [39,52] in both control and *PARKIN*^S65N/S65N^ patient fibroblast lines. To induce substantial levels of mitophagy in culture, patient and control cells expressing *mito*-QC were stimulated with A/O for 24 h. Upon A/O stimulation, we observed no difference in mitophagy between control and *PARKIN*^S65N/S65N^ *mito*-QC cells (electronic supplementary material, figure S8). These data are consistent with a previous report demonstrating that endogenous Parkin is dispensable for basal and depolarization-induced mitophagy in patient-derived primary fibroblasts [52].

#### 2.5.4. Structural analysis of the Parkin^S65N^ mutant protein

To understand precisely how the pathogenic S65N mutation affects Parkin activity, we crystallized and solved the structure of the novel human Parkin PD mutant (UBLR0RBR, Δ84–143) at 2.9 Å. Comparative analyses of wild-type and Parkin^S65N^ x-ray crystal structures did not reveal any conformational differences between the wild-type and mutant protein (figure 5*e* and electronic supplementary material, figure S6*c*), indicating that the S65N mutation does not significantly perturb Parkin structure. However, as the S65N mutation prevents incorporation of a phosphate group at this position, the absence of a negative charge at the UBL-IBR interface inhibits the ability of Parkin^S65N^ to adopt the open conformation required for full E3 activity. This contrasts to wild-type Parkin, where phosphorylation at Ser65 induces key conformational changes and new interactions [53–56].

## 3. Discussion

Combining a novel mouse knock-in model and unique human clinical case studies, we present the most compelling evidence to date demonstrating the physiological and clinical significance of PINK1-dependent Parkin Ser65 phosphorylation. Furthermore, despite over 20 putative substrates reported in the literature [57,58], our research indicates that inactivation of this PINK1 phosphorylation site on Parkin alone is sufficient for humans to develop early onset Parkinson's disease (PD).

Understanding the mechanistic basis of DA neuronal loss in mammalian models of PINK1 and Parkin has proved challenging, because it has been difficult to recapitulate a PD-like neurodegenerative phenotype in rodents, with the notable exceptions of mutant *Parkin* over-expression in mice and rats [59,60]; the *Pink1*-null rat model [61]; and exposure of *Parkin*-null mice to chronic mitotoxic stress [21]. While *Parkin*^S65A/S65A^ mice have an anatomically intact nigrostriatal pathway with no detectable loss of viability, denervation or dopamine depletion even after ageing for 18 months (figure 4*a*–*c*), we observed selective locomotor dysfunction particularly on balance beam performance (figure 3*a*). Our results are consistent with previous reports of motor deficits reported in *Parkin* knockout mice [17] and *Pink1* knockout mice [29] in the absence of nigrostriatal pathology.

The neurobiological basis of the fine motor defects emerging in *Parkin* and *Pink1* genetic models is poorly understood. We did not observe any substantial changes in the absolute levels of dopamine in *Parkin* knock-in mice. Interestingly, multiple lines of evidence suggest that *Parkin* knockout mice exhibit compartment defects with reduced striatal synaptic release of dopamine, associated with reduced expression of both the dopamine transporter (DAT) and vesicular monoamine transporter (VMAT2) and higher levels of extracellular dopamine in the striatum [17,18]. Mild mitochondrial defects have also been reported in 24-month old *Parkin* knockout mice that are not observed in younger mice (9 months of age) [20]. These effects were restricted to the striatum, and not detected in other brain regions including the midbrain or cortex [20]. Using a similar approach, our analysis revealed a mild, age-dependent defect in mitochondrial respiration in the striatum of *Parkin* knock-in mice (figure 3*b*). Interestingly, this was not associated with any change in basal nigrostriatal mitophagy in *Parkin* knock-in mice (figure 4*d*,*e*). Our current findings are also consistent with our recent demonstration of PINK1-independent mitophagy *in vivo* [39]. Recent analysis of *Drosophila* *melanogaster* models expressing fluorescent mitophagy reporters has also revealed that basal mitophagy proceeds independently of both PINK1 and Parkin *in vivo* [38]. Nevertheless, because cell culture studies *in vitro* provide a compelling case for Parkin activation in stress-evoked mitochondrial quality control pathways [4–6], it will be vital to investigate if nigrostriatal mitophagy is specifically affected in models of constitutive mitotoxicity reporting PD-relevant pathology, e.g. Pol*γ* mutator mouse crossed with *Parkin* knockout mice [21,62]. Ultimately, our findings highlight the importance of uncovering the mitophagy-independent pathways by which endogenous Parkin sustains striatal function and mitostasis during ageing. Our data also adds to the growing body of evidence that suggests multiple mitophagy pathways operate to sustain mitochondrial quality control during specific contexts i.e. under basal conditions compared to extreme or chronic stress.

At the molecular level, the feed-forward mechanism of Parkin activation stipulates that (i) PINK1-dependent generation of phospho-ubiquitin stimulates Parkin recruitment to damaged mitochondria, (ii) this primes Parkin for phosphorylation by PINK1 and thus maximal activation, (iii) leading to further phospho-ubiquitin accumulation [4–6]. Evidence for this model includes the demonstration that the Parkin His302Ala (H302A) mutant, that cannot bind phospho-ubiquitin but is catalytically competent, exhibits reduced mitochondrial recruitment, Parkin Ser65 phosphorylation and ubiquitylation of substrates in HeLa cells [11,41]. However, HeLa studies also suggest that the initial amount of phospho-ubiquitin generated by PINK1 (independent of Parkin activity) is likely to be low because over-expression of the catalytically inactive Parkin Cys431Ser mutant does not result in significantly detectable phospho-ubiquitin accumulation [12,15]. Consistent with this latter observation, we did not observe any phospho-ubiquitin accumulation in mature primary cortical neurons (figure 2) or adult primary lung fibroblasts (electronic supplementary material, figure S3) subjected to mitochondrial depolarization from *Parkin*^S65A/S65A^ and *Parkin* knockout mice. This is consistent with recent analysis of CRISPR/Cas9-mediated S65A mutant human ES cell-derived neurons stimulated with A/O [25]. By contrast, we did detect phospho-ubiquitin accumulation in Parkin^S65N^ mutant patient-derived fibroblasts and HeLa cells (figure 5*c* and electronic supplementary material, figure S6*b*), albeit at lower levels than wild-type cells, suggesting potential redundancy of Parkin in these cell types with both Parkin-dependent and Parkin-independent formation of phospho-ubiquitin. The MUL1 E3 ligase has recently been reported to operate in a parallel pathway to Parkin in non-neuronal cells, and could account for the phospho-ubiquitin we observe in our human cell systems [63], although further work is required to substantiate this.

We and others have previously demonstrated that phospho-ubiquitin can partially activate recombinant non-phosphorylatable Parkin S65A protein *in vitro* [8,12]. Despite detectable accumulation of phospho-ubiquitin in fibroblasts, we observed complete loss of E3 activity in *PARKIN*^S65N/S65N^ mutant cells. The interaction affinity between the non-phosphorylated Parkin-phospho-ubiquitin complex with its charged E2 ∼ Ub is approximately 20-fold lower than that for the Phospho-Ser65-Parkin and phospho-ubiquitin complex [64], which may partly explain the lack of activation of endogenous Parkin^S65N^ (and similarly Parkin^S65A^) protein. Our results therefore highlight the importance of studying signalling pathways at endogenous levels in physiologically relevant systems. Moreover, our data may also be explained by recent structural analysis of Parkin activation mechanisms. Initial binding of phospho-ubiquitin triggers a conformational shift that exposes a donor ubiquitin-binding site and partial activation of Parkin catalytic activity [54]. The subsequent PINK1-dependent phosphorylation of Parkin Ser65 ultimately drives Parkin activation by a series of structural alterations. In particular, displacement of the phospho-Ubl domain maintains interaction of the ubiquitin-charged E2 to the RING1 domain, and the phospho-Ubl subsequently binds to a basic phosphate-binding site on RING0 (flanked by residues Lys161 and Lys211 as well as Arg163) [53,55,56]. The binding of phospho-Ubl to RING0 leads to the release of the catalytic RING2 domain thereby exposing C431 to promote full Parkin activation and enhancing interactions with E2 [53,55,56]. Collectively, our endogenous neuronal results combined with recent structural insights highlight the pre-eminent status of Parkin Ser65 phosphorylation in modulating its catalytic activation.

The clinical importance of endogenous PINK1-mediated Parkin phosphorylation is substantiated by our discovery of two unrelated PD patients harbouring novel mutations at Parkin Ser65. Numerically, mutations in Parkin are associated with early onset PD, accounting for approximately 40% of cases with age of onset below 45 years; however, patients with pathogenic Parkin inactivating mutations have been reported with mid-age of onset that would be consistent with the second case [65]. In line with our mouse data, we found that basal and depolarization-induced mitophagy in S65N patient-derived primary fibroblasts was not diminished, despite a loss of Parkin E3 ligase activity (electronic supplementary material, figure S8). In the future, it will be exciting to address whether this is the case in a more neural context, by generating dopamine neurons derived from human pluripotent stem cells (hPSCs).

Although PINK1–Parkin signalling constitutes a clear pathway that modulates depolarization-induced mitophagy *in vitro*, basal mitophagy is unaffected in both *Pink1* knockout and *Parkin* S65A knock-in mice. Though the clinical significance of PINK1–Parkin signalling is incontrovertible, the contribution of this pathway to mitophagy *in vivo* remains unclear, as does the contribution of dysregulated mitophagy to PD [66]. We predict that distinct mitophagy pathways orchestrate mitochondrial homeostasis under steady-state and stress-response scenarios.

In summary, this study is the first to establish both the physiological and clinical significance of PINK1-mediated phosphorylation of Parkin at serine 65. Our findings shed much-needed light on endogenous Parkin function and should provoke a reassessment of how it orchestrates cell-specific mitostasis *in vivo*.

## 4. Material and methods

### 4.1. Generation and validation of *Parkin*^S65A/S65A^ knock-in mice

*Parkin*^S65A/S65A^ mice were generated by TaconicArtemis GmbH, using a targeting strategy to introduce a constitutive knock-in of a point mutation (KI-PM) in the *Park2* gene by homologous recombination in mouse ES cells. The targeting strategy was based on NCBI transcript NM_016694.3. Exon 1 contains the translation initiation codon, and the S65A mutation was introduced into exon 3. A positive selection marker (puromycin resistance, PuroR) flanked by FRT sites was inserted into intron 2. The targeting vector was generated using BAC clones from the C57BL/6 J RPCIB-731 BAC library and transfected into the TaconicArtemis C57BL/6N Tac ES cell line. Homologous recombinant clones were isolated using positive and negative (thymidine kinase, TK) selection, and the constitutive KI-PM allele was obtained after Flp-mediated removal of the selection marker. This KI-PM allele was predicted to express the mutated *Park2* S65A protein product. The remaining recombination site will be located in a non-conserved region of the genome. The knock-in mice were generated and maintained on an inbred C57BL/6J background. Genotyping was performed by endpoint PCR using genomic DNA isolated from tails using the following primer sets at an annealing temperature of 60°C: Primer 1 (5′GAACAAGATAGGAGCACAGAAGG 3′) and Primer 2 (5′GACCAATTTACCTCTCGAGTGC 3′) that enabled detection of the wild-type (290 bp) and S65A knock-in (365 bp) alleles. The *mito*-QC mouse model used in this study was generated as previously described on a C57BL/6N background strain [22] and crossed with *Parkin* S65A knock-in heterozygote animals to produce homozygous knock-in animals and wild-type littermate controls with endogenous *mito*-QC reporter.

### 4.2. Materials and reagents

HaloLink resin was purchased from Promega. All mutagenesis was carried out using the QuikChange site-directed mutagenesis method (Stratagene) with KOD polymerase (Novagen). All DNA constructs were verified by the MRC Protein Phosphorylation and Ubiquitylation Unit (PPU) DNA Sequencing Service, School of Life Sciences, University of Dundee, using DYEnamic ET terminator chemistry (Amersham Biosciences) on Applied Biosystems automated DNA sequencers. DNA for bacterial protein expression was transformed into *Escherichia coli* BL21 DE3 RIL (codon plus) cells (Stratagene). Stock solutions of carbonyl cyanide m-chlorophenyl hydrazone (CCCP; Sigma), antimycin A (Sigma) and oligomycin (Sigma) were used for experiments in cells. Unless otherwise specified, general reagents and chemicals were from Sigma-Aldrich (Merck) and cell culture reagents were from Gibco/Invitrogen (Thermo Fisher Scientific). All cDNA plasmids, antibodies and recombinant proteins generated in house for this study are available on request through our dedicated reagents website: https://mrcppureagents.dundee.ac.uk/

### 4.3. Mouse motor and behavioural studies

In total, 51 *PARK2 S65A* mice were used for behavioural experiments in groups which consisted of 25 wild-type (14 males and 11 females) and 26 *Park2*^S65A/S65A^ mice (11 males and 15 females). At 18 months 35 mice remained, 16 wild-type (10 males and 6 females) and 19 *Park2*^S65A/S65A^ mice (9 males and 10 females). Mice were housed in sex-matched littermate groups of between two and five animals per cage with lights adjusted to a 12 L : 12 D cycle (lights on at 06.30) and with a constant ambient room temperature of 21° ± 1°C. Animals had *ad libitum* access to food and water throughout. The mice underwent behavioural testing at 12 months and 18 months of age and all experiments were conducted in accordance with the 2013 European Union Directive 2010/63/EU and the UK Animals (Scientific Procedures) Act of 1986 and local ethical review at the University of Cardiff.

The motor test battery has been described previously elsewhere [67], but briefly mice were tested for simple neurological deficits using: clasping and reaching assays; accelerating rotarod and balance beam test to measure motor coordination and balance; 30 min and 24 h open field activity; footprint analysis to measure changes in gait; and inverted grid task to measure grip strength. For the rotarod test (Ugo Basile, Varese, Italy), mice are placed on a rotating beam which accelerates. Initial training consisted of training the mice at different speeds for 2 min (4, 8 and 12 r.p.m.). Data were collected from five sessions at the 12-month time point and two sessions at the 18-month time point whereby the mice were placed on the rod that accelerated from 4 to 40 r.p.m. over 5 min. The latency of the mice to fall from the beam was recorded and used as a measure of motor coordination. For the balance beam test (see above reference for dimensions), each mouse was trained to run the beam prior to the test session. This consisted of starting the mice in the goal box at the top of the inclined beam and over repeated exposures moving the starting point on the beam to the lower end (start point) such that the mice ran further as the training progressed. Training was complete when the mouse was placed on the lower end of the beam (facing away from the beam) and the mouse turned and ran the length of the beam unprompted. Foot-slips were recorded when a foot slipped from the beam onto the narrow ledge that ran either side of the length of the beam. Each mouse provided data (fore and hind limb foot-slips, latency to turn and cross the beam) from two runs for analysis. Gait analysis was run using a footprint test whereby the feet of the mice were painted with red (front feet) and blue (rear feet) non-toxic water-based paint, and the mice were permitted to run a 1-m corridor that was fitted with paper on the floor such that they left coloured paw prints. The paw print patterns permit the measurement of variation in gait: stride length; base width (distance between left and right front/hind limbs); front/hind paw overlap (in normal locomotion the hind paw placement overlaps closely with the location where the fore paw had previously landed; gait impairment is manifest by an increase in the magnitude and variability of the separation. For the motor activity testing using an automated system (Med Associates Inc, Vermont, USA), mice were placed in an open field arena for 32 h with free access to food and water throughout. Data were collected automatically with activity being measured as the number of infrared beam breaks. Activity was measured as the initial 30 min period (transfer activity) and a 24 h period (15.00 to 15.00) to capture a circadian cycle. In addition, several simple behavioural assessments were run: clasping and reaching reflexes where the mice is suspended by the tail and lowered to the benchtop with clasping and reaching measured through observation and recorded as present or absent; negative geotaxis, measuring the natural inclination of the mouse to face upwards when placed head down on a vertical grid, with the mouse being allowed 30 s to correct its orientation to the upright position; and horizontal grid grip strength, whereby the mouse is placed on a flat grid surface which is then rotated 180° along the horizontal axis such that the mouse is clinging to the underside of the grid, the maximum time for the mouse to remain upturned being 60 s without falling.

#### 4.3.1. Statistical analyses of mice behavioural studies

Parametric split-plot analyses of variance using age as the within groups factor and genotype and sex as between-group factors were used where applicable as three-way ANOVAs. All statistical analyses were conducted on Genstat 17 (VSN International, Hemel Hempstead, UK). Missing values were corrected with an inbuilt linear interpolation routine. For non-parametric data, *χ*^2^ was used to determine significance. Significance level was taken at 95% confidence.

### 4.4. Immunohistochemistry, iDISCO^+^ and microscopy

Transcardial perfusion was performed under terminal general anaesthesia to obtain tissues for histological analyses. Histology, immunohistochemistry, immunocytochemistry and confocal microscopy were performed as previously described [22,39,68,69], with minor modifications. Following terminal anaesthesia via intraperitoneal administration of Euthatal, adult animals were trans-cardially perfused with phosphate-buffered saline (PBS) to remove excess blood. Tissues were rapidly harvested and processed by immersion fixation in freshly prepared 3.7% PFA at pH 7.0 in 0.2 M HEPES. For *mito*-QC animals, transverse brain sections (200 mm) were acquired using a vibratome (Leica). For chromogenic immunohistochemistry and stereological analyses, coronal brain sections (40 mm) were cut on a sledge microtome equipped with a freezing stage (Leitz) and processed for free-floating immunohistochemistry. The following primary antibodies were used: rat monoclonal anti-lysosomal associated membrane protein 1 (LAMP1) (1D4B) (Cat#: sc-19992 RRID: AB_2134495; Santa Cruz Biotechnology, Inc.); rabbit, sheep and mouse anti-Tyrosine Hydroxylase (rabbit: Cat#: AB152; RRID: AB_390204; sheep: Cat#: AB1542; RRID: AB_90755; mouse: Cat#: MAB318; RRID: AB_2201528; Millipore); rabbit polyclonal anti-Ionized calcium binding adaptor molecule 1 (Iba1) (Cat#: 019-19741; RRID: AB_839504 - Wako), chicken anti-GFP (Cat# GFP-1020, RRID:AB_10000240; Aves Labs; Cat# ab13970, RRID:AB_300798; Abcam). Alexa-Fluor conjugated secondary antibodies were obtained from Life Technologies (Molecular Probes/Thermo Fisher Scientific) as described previously [39]. VECTASHIELD Antifade Mounting Medium H-1000 was used to mount tissue sections on slides (Leica Surgipath). For cell counts and tinctorial histochemistry, Cresyl fast violet (Nissl) stain was obtained from Sigma-Aldrich (C5042-10G). For whole-mount imaging of the nigrostriatal pathway in intact mouse brains, specimens were processed, stained using rabbit-TH (AB152) and optically cleared using the detailed protocol specified for iDISCO^+^ [33] (https://idisco.info). Following the acquisition of images from intact whole brains (dorsal and ventral surfaces), cleared brain specimens were hemisected along the midline and subsequently bisected to facilitate deeper imaging in the sagittal and coronal planes, respectively. Images were acquired using a Zeiss LSM 710 laser scanning microscope (Plan-Neofuar 340 objective, NA 1.30; Plan Apochromat 363 objective NA 1.4; Plan Apochromat 320 objective, NA 0.8), or a Zeiss LSM880 Airyscan confocal scanning microscope (ZEISS; Plan Apochromat 363 objective, NA 1.4) and processed using ZEISS Zen software/Adobe Photoshop or Imaris (Bitplane) for 3D isosurface rendering. Images were digitally altered within linear parameters, with minimal adjustments to levels and linear contrast applied to all images.

### 4.5. Quantification and statistical analysis

Semi-automated quantitation images were processed with Volocity 6.3 image analysis software (PerkinElmer) using algorithms developed to analyse object overlap and count individual structures. For all analyses, we obtained images using uniform random sampling by an experimenter blind to all conditions. All images in each experimental group were processed as a batch using identical protocols. All images were prefiltered to suppress noise (3 × 3 median filter). The same strategy was used to quantify all non-immunolabelled mito-QC-labelled images using auto-thresholding to identify objects (mean intensity + 3 standard deviations). Objects were filtered using a minimum size cutoff (0.16 mm^2^) and touching objects were separated using a guide size (0.4 mm^2^). Mitolysosomes were typically identified on the basis of thresholded red signal not overlapping with green (this condition was relaxed in the case of samples where dequenching of the *mito*-QC probe was observed). Where dequenching or spectral-overlap was observed in some instances, we employed LAMP1 immunohistochemistry to verify the lysosomal nature of mitolysosomes and green channel/combined with LAMP1 immunostaining was used as a proxy for mitolysosomes. In the case of immunolabels used to identify cells of interest in brain sections (TH, Iba1), we employed an autothresholding criterion of mean intensity + 1.5 standard deviations. A minimum size of 0.16 mm^2^ was required, and a ‘Fill Holes in Objects' processing step was applied.

Cell counts were conducted using two-dimensional stereology with an automated stage and stereology module ‘NEWCAST TM’ from Visiopharm integrator system software (Visiopharm) on an Olympus Bx50 microscope (Olympus Optical Co. Ltd, Tokyo, Japan). For NeuN immunohistochemistry, cell counts were conducted on a 1 : 12 series throughout the entire striatum (left and right sides), with the counting objective set at 100× and the randomized sampling counting frame area at 285 µm^2^ and corrected using the Abercrombie formula [70]. For the TH and Calbindin double labelling, cell counts were carried out on a 1 : 12 series through the substantia nigra with the counting objective set at 40×, and all visible cells were counted within this area with double-stained cells denoting A10 dopamine cells and cells only stained for TH denoted as A9 dopamine cells.

### 4.6. Mitochondrial respiration assays

Wild-type and *Parkin* S65A knock-in mice (10 per genotype) were killed by decapitation without prior anaesthesia. A fragment of the tail was retrieved for confirmation of the genotype, determined by PCR amplification, as previously described [71]. The brains were rapidly removed and the regions of interest (striatum, midbrain and cortex) dissected on ice and weighed. Post-nuclear supernatants were obtained by manual homogenization of brain samples in nine volumes of isolation buffer (300 mM sucrose, 1 mM EGTA, 5 mM Tris–HCl pH 7.4), using a glass Dounce homogenizer with a glass pestle, followed by centrifugation at 1000*g*, 4°C, for 10 min. The post-nuclear supernatants were directly used for respiration and protein assays, as well as for the analysis of citrate synthase (CS) activity. The post-nuclear supernatants were analysed in a thermostatically controlled oxygraphic chamber at 25°C with continuous stirring (Oxygraph-2k, Oroboros Instruments, Innsbruck, Austria) as previously described [20]. In brief, the samples were resuspended in 2 ml of mitochondrial respiration medium (100 mM KCl, 40 mM sucrose, 10 mM TES, 5 mM MgCl_2_, 1 mM EGTA, 0.4% w/v fatty acid free-BSA (bovine serum albumin), pH 7.2) and the protein concentration was determined (Bradford protein assay, Bio-Rad). Each respiration assay was run with a constant amount of protein from one wild-type and one knock-in mouse (0.3 mg for striatum, 0.6 mg from midbrain and cortex). The results were expressed in nmol of O_2_/min mg^−1^ of total protein. The respiration rate was recorded sequentially: (i) *basal respiration* in the presence of complex I substrates (10 mM glutamate, 5 mM malate); (ii) *state 3 respiration* with addition of 1 mM ADP; (iii) *state 4 respiration* with 1 µg ml^−1^ oligomycin; and (iv) *maximal respiration rate* was determined by progressive additions of 1.25 µM carbonyl cyanide m-chlorophenyl hydrazone (CCCP). The *respiratory reserve capacity* was calculated as maximal respiration − basal respiration. *Respiratory control ratio or RCR* was calculated as state 3/state 4.

### 4.7. Determination of citrate synthase activity

CS activity was determined using a colorimetric assay based on the reaction between 5,5′-dithiobis-(2-nitrobenzoic acid) (DTNB) and CoA-SH to form 5-thio-2-nitrobenzoic acid (TNB), which exhibits maximum absorbance at 412 nm, according to the manufacturer's instructions (Sigma, CS0720). In brief, 20 µl of cold CelLytic M supplemented with protease inhibitors (protease inhibitor cocktail, Thermo Scientific 1861279) was added to 10 µl of post-nuclear supernatant. The sample was homogenized and centrifuged at 12 000*g* for 10 min. The supernatant was collected, and protein concentration was determined by the Bradford method (BioRad). Fifteen to 20 µg of protein was added to a 200 µl reaction mixture containing 1× assay buffer, 300 µM acetyl CoA and 100 µM DTNB. The reaction was initiated by addition of oxaloacetate (OAA) to a final concentration of 0.5 mM. CS activity was evaluated by following the absorbance of the reaction mixture at 412 nm (SpectraMax M4) before and after addition of OAA every 10 s, for 2 min. Each sample was run in duplicate and CS activity was expressed as units/min mg^−1^ of total protein.

### 4.8. Dopamine HPLC analysis

Dopamine and its metabolite 3,4-dihydroxyphenylacetic acid (DOPAC) concentrations were measured in the dorsal striatum by reverse phase high-performance liquid chromatography coupled with electrochemical detection (Coulochem III, Thermo Scientific) as described earlier [72]. Briefly, 18-month-old wild-type and *Parkin*^S65A/S65A^ mice were killed by cervical dislocation. Brains were quickly removed, pre-commissural dorsal striatum was dissected on an ice-cold Petri dish and freshly homogenized in 0.4 M percloric acid. Following centrifugation and filtration of the supernatant, dopamine and DOPAC were separated using a C18 reverse phase column (Thermo Scientific). The mobile phase consisted of 10% acetonitrile, 75 mM NaH_2_PO_4_·H_2_O, 0.17 mM octanesulfonic acid, 2.5 mM triethylamine and 25 mM EDTA, adjusted to pH 3.0 with orthophosphoric acid, and was delivered at a flow rate of 0.6 ml min^−1^. Protein levels were determined by Lowry assay from pellets of the homogenates and catecholamine concentrations are expressed per milligram protein.

### 4.9. Antibodies for biochemical studies

The following primary antibodies were used: total Parkin immunoblotting (sc32282, Santa Cruz), mouse Parkin immunoprecipitation (in-house, S328D, 5th bleed), βI-tubulin (Sigma), β-actin (Sigma), Calbindin (c9848Sigma), GAPDH (Santa Cruz), CISD1 (Proteintech), NeuN (MAB377, Millipore), PINK1 immunoblotting (BC100-494, Novus), PINK1 immunoprecipitation (in-house S085D, 3rd bleed), Mitofusin2 (MFN2) (in-house, S781D, 4th bleed), OPA-1 (#612607, BD Biosciences), RHOT2 (Miro2) (H00089941, Abnova), COX IV (#4850, Cell signalling technology), PDH (#2784, Cell signalling technology), Tyrosine Hydroxylase (ab112, Abcam), VDAC1 (ab15895, Abcam), Vinculin (#4650, Cell signalling technology). Horseradish-peroxidase (HRP)-conjugated secondary antibodies (Sigma) were used. Anti-Parkin phospho-Ser65 rabbit monoclonal antibody was raised by Abcam in collaboration with the Michael J. Fox Foundation for Research. Anti-phospho-Ser65-ubiquitin rabbit polyclonal antibodies were generated by 21st Century Biochemicals Inc. (Marlborough, MA, USA) using two phosphorylated peptides, C-Ahx-YNIQKE[pS]TLHLVL-amide and Ac-YNIQKE[pS]TLHLVL-Ahx-C-amide, that correspond to amino acid residues 59–71 of ubiquitin. Antibodies were affinity purified using phosphorylated and non-phosphorylated versions of the peptide immunogen (electronic supplementary material, figure S9*a*). A fragment of human Miro1 fusion protein encompassing residues 1–592 with N-terminal His-SUMO tag was used as an immunogen to raise a sheep polyclonal anti-Miro1 antibody (S531D). Antibodies were affinity purified from antisera and characterized indicating cross-reactivity with Miro1/2 as described in the electronic supplementary material, figure S9*b*.

### 4.10. Tissue culture: proliferating and primary cells

Flp-In T-Rex HeLa stable cell lines were cultured in DMEM (Dulbecco's modified Eagle's medium) supplemented with 10% FBS (fetal bovine serum), 2 mM l-glutamine, 1× penicillin/streptomycin 1× non-essential amino acids (Life Technologies), 15 µg ml^−1^ of blasticidin and 100 µg ml^−1^ of hygromycin. Cell transfections of untagged wild-type or mutant Parkin were performed using polyethylene method [73]. Cultures were induced to express protein by addition of 0.1 µg ml^−1^ of doxycycline to the medium for 24 h. To depolarize or uncouple mitochondrial membrane potential, proliferating cells were treated with 10 µM antimycin (Sigma) and 1 µM oligomycin dissolved in DMSO. Primary mouse cortical neurons were isolated from the brains of wild-type or mutant embryos of either sex at E16.5. Embryonic cortices were collected in HBSS, and cells were dissociated by incubation with trypsin-EDTA (#25300-054, Gibco) at 37°C. Cells were then diluted in Neurobasal medium containing B27 supplement, Glutamax, penicillin/streptomycin and plated at a density of 6.0 × 10^6^ cells/well on 6-well plates coated with 0.1 mg ml^−1^ poly-l-lysine (PLL; Sigma). Neurons were cultured at 37°C in a humidified incubator with 5% CO_2_. Every 7 days, the medium was replaced with fresh medium containing B27. To depolarize or uncouple mitochondrial membrane potential in neurons, cultures were treated with 10 µM antimycin (Sigma) and 1 µM oligomycin dissolved in DMSO at 37°C.

Primary lung fibroblasts were derived from adult mice as previously described and maintained in DMEM/20%FBS/penicillin–streptomycin at 37°C/5% CO_2_ [26]. Primary human skin fibroblast cultures at a low passage from the patient and one matched control were established from a minimally invasive skin biopsy sample according to routine culture procedures. The cells were cultured in 5% CO_2_ and 90% humidity in high-glucose DMEM media (Gibco) supplemented with 10% FBS (LSP #S-001-BR), 1× non-essential amino acids, 1 mM sodium pyruvate, 100 Units/ml penicillin, 100 µg ml^−1^ streptomycin and 2 mM l-glutamine. The retrovirus for analysis of *mito*-QC in human fibroblasts was generated as previously described [52]. The cDNA for the *mito*-QC reporter (mCherry, GFP and residues 101–152 of human FIS1) was cloned into a pBABE.hygro vector or pBABE.puro vector. The construct was co-transfected into 293FT cells with GAG/POL and VSV-G expression plasmids (Clontech, Saint-Germain-en-Laye, France) for retrovirus production using Lipofectamine 2000 (Life Technologies) in accordance with the manufacturer's instructions. Virus was harvested 48 h after transfection and applied to cells in the presence of 10 mg ml^−1^ polybrene. Cells were selected with either 500 µg ml^−1^ hygromycin B or 2 µg ml^−1^ puromycin. Selected cells were seeded and incubated for 24 h into glass bottom dishes (Ibidi μ-Dish 35 mm) with media free of sodium pyruvate and non-essential amino acids. Mitophagy was stimulated for 24 h with 10 µM antimycin A (Sigma #A8674) and 5 µM oligomycin A (Sigma #75251), and controls were treated at equal volumes of DMSO. Cells were fixed for 20 min with 3.7% PFA (Sigma #P6148) pH 7.0, and subsequently washed twice with 2 ml of DMEM + 10 mM HEPES pH 7.0 and incubated for at least 10 min. Images were acquired using a ZEISS LSM 710 confocal microscope and quantified using Volocity 6.3 image analysis software as previous described [39].

### 4.11. Tissue and cell lysis

For analysis of endogenous Parkin protein expression *in vivo*, fresh brains were rapidly excised, placed in cold PBS and microdissected with ultrafine microknives under stereomicroscopy. Upon isolation, each brain region was collected in a single 1.5 ml microcentrifuge tube and immediately plunged into dry ice. Tissue samples were stored at −80°C until ready for processing. To make protein extracts, all tissues (neural subregions and extra-neural tissues) were weighed and defrosted on wet ice in fivefold mass excess of freshly prepared, ice-cold lysis buffer containing: 50 mM Tris/HCl pH 7.5, 1 mM EDTA pH 8.0, 1 mM EGTA pH 8.0, 1% Triton X-100, 0.25 M sucrose, 150 mM NaCl, 2 mM sodium orthovanadate, 1 mM NaF, 10 mM sodium glycerolphosphate, 1.15 mM sodium molybdate, 4 mM sodium tartrate dehydrate, 100 mM 2-chloroacetamide, 1 mM DTT and complete protease inhibitor cocktail (Roche). All inhibitors and DTT were added immediately prior to use. Tissue homogenization was performed using a probe sonicator at 4°C (Branson Instruments). Crude lysates were incubated at 4°C on wet ice for 30–45 min, before clarification by centrifugation at 14 000 r.p.m. for 30 min at 4°C. Supernatants used for subsequent steps were carefully removed and either used for downstream biochemical analyses or snap-frozen and stored at −80°C.

Primary neurons or Hela cells were sonicated in lysis buffer containing 50 mM Tris–HCl (pH 7.5), 1 mM EDTA, 1 mM EGTA, 1% (w/v) Triton, 1 mM sodium orthovanadate, 10 mM sodium glycerophosphate, 50 mM sodium fluoride, 10 mM sodium pyrophosphate, 0.25 M sucrose, 1 mM benzamidine, 0.1 mM PMSF and protease inhibitor cocktail (Roche). Following the sonication, lysates were incubated for 30 min on ice. Samples were spun at 20 800*g* in an Eppendorf 5417R centrifuge for 30 min. Supernatants were collected and protein concentration was determined using the Bradford kit (Pierce). Primary human skin fibroblasts were lysed with ice-cold lysis buffer containing 50 mM Tris–HCl pH 7.5, 1% (v/v) Triton X-100, 1 mM EGTA, 1 mM sodium orthovanadate, 50 mM sodium fluoride, 10 mM β-glycerophosphate, 5 mM sodium pyrophosphate, 0.1 µg ml^−1^ microcystin-LR (Enzo Life Sciences), 270 mM sucrose, complete EDTA-free protease inhibitor cocktail (Sigma Aldrich Cat # 11836170001). Lysates were centrifuged at 20 800*g* for 10 min at 4°C. Supernatants were collected, quantified by Bradford assay (Thermo Scientific) and subjected to immunoblot analysis.

### 4.12. Membrane fraction enrichment

Cells were collected in ice-cold PBS containing 200 mM chloroacetamide. They were then lysed in buffer containing 250 mM sucrose, 20 mM HEPES, 3 mM EDTA, 1% (w/v) 1 mM sodium orthovanadate, 10 mM sodium β-glycerophosphate, 50 mM NaF, 5 mM sodium pyrophosphate, pH 7.5 and protease inhibitor cocktail (Roche) supplemented with 100 mM chloroacetamide at 4°C. Cells were disrupted using a glass hand-held homogenizer (40 passes) and the lysates were clarified by centrifugation (800*g* at 4°C for 10 min). The supernatant was harvested and subjected to an additional centrifugation step at 16 600*g* for 10 min at 4°C. The resulting pellet containing the mitochondrial-enriched fraction was resuspended in the above buffer containing 1% Triton X-100 and centrifuged at 13 000 r.p.m. for 10 min at 4°C. The supernatant arising from this step contained solubilized mitochondrial proteins.

### 4.13. Immunoblotting and immunoprecipitation

For immunoprecipitation of endogenous PINK1 from human skin fibroblasts, 150 µg of whole-cell lysate was incubated overnight at 4°C with 5 µg of protein G pre-bound to PINK1 antibody. The immunoprecipitations were washed four times with lysis buffer containing 150 mM NaCl, then twice with buffer containing 50 mM Tris–HCl pH 7.5, 0.1 mM EGTA, and eluted by resuspending in 20 µl of 1× SDS sample buffer. Denatured samples were subjected to SDS-PAGE (4–12% Bis-Tris gels) and were transferred on to Protran 0.2 NC nitrocellulose membranes (Amersham). Membranes were blocked for 1 h at room temperature with 5% non-fat milk (Marvel) or BSA in TBST (Tris-buffered saline (50 mM Tris/HCl and 150 mM NaCl, pH 7.5) containing 0.1% Tween-20) in PBS pH 7.4 and incubated with the indicated antibodies overnight at 4°C with agitation. Detection was performed using HRP-conjugated secondary antibodies (Goat anti-Rabbit IgG (H + L) Secondary Antibody, HRP conjugate, #31460, Thermo Scientific; Rabbit anti-Mouse IgG (H + L) Secondary Antibody, HRP conjugate, #31450, Thermo Scientific; Rabbit anti-Sheep IgG (H + L) Secondary Antibody, HRP conjugate, #31480, Thermo Scientific). Membranes were developed using standard chemiluminescence with ECL (Amersham) and exposure to hyperfilm (GE Healthcare).

### 4.14. Activity-based profiling of Parkin

Transthiolation activity of Parkin activity in primary human skin fibroblasts was measured as described previously [41]. Briefly, Parkin probe (produced by Dr. Satpal Virdee, MRC PPU) was added to 50 µg of whole-cell lysate at a final concentration of 5 µM. Reactions were incubated at 30°C for 4 h and quenched by the addition of LDS sample buffer with β-mercaptoethanol, before analysis via SDS-PAGE (4–12% Bis-Tris Gels, Novex) and immunoblotting as previously described.

### 4.15. Ubiquitin enrichment

His-Halo-Ubiquilin UBA-domain tetramer (HALO-UBA^UBQLN1^) was expressed in *E. coli* BL21 cells, affinity purified on Ni-NTA-agarose and dialysed into 50 mM HEPES pH 7.5, 10% glycerol, 150 mM NaCl, 1 mM DTT [11]. For ubiquitin capture, 200–400 µg of whole-cell extract or 1 mg of membrane-enriched fraction was used for pulldown with HALO-UBA^UBQLN1^ [11]. HALO-UBA^UBQLN1^ was incubated with 200 µl of HaloLink resin (Promega) in binding buffer (50 mM Tris–HCl pH 7.5, 150 mM NaCl, 0.05% NP-40) overnight at 4°C under agitation [11]. HALO-TUBE beads (20 µl) were added to lysates and incubated at 4°C for 4 h under agitation. Beads were washed three times with lysis buffer containing 0.25 M NaCl, and eluted by resuspending in 20 µl of 1× LDS sample buffer containing 1 mM DTT.

### 4.16. Protein purification, crystallization and structure determination

Parkin S65N (Parkin^S65N^) (Δ84–143) was cloned into pET156P vector (DU56345) and expressed as His-SUMO fusion in *E. coli* BL21 cells, using conditions similar to those described in Chaugule *et al.* [74]. His-tagged Parkin^S65N^ was purified using Ni-NTA resin. Protein eluted from Ni resin was incubated overnight at 4°C with SENP1 protease to remove the His-SUMO tag, and dialysed in 20 mM Tris pH 7.5, 75 mM NaCl and 250 µM TCEP buffer. Dialysed protein was further incubated with Ni-NTA resin and tag-free Parkin^S65N^ was collected in the flow through, purified on Mono-Q and Superdex-75 columns (as described in [64]) and concentrated to 8 mg ml^−1^ for crystallization. Parkin^S65N^ was crystallized at 4°C in 100 mM BIS-TRIS pH 5.5, 200 mM LiSO_4_ and 20% PEG3350 by using a vapour diffusion method under sitting drop. Crystals were flash frozen in 20% glycerol, the native dataset was collected at ID30A-1 beamline, European Synchrotron Radiation Facility (ESRF, Grenoble). Data were processed using Mosflm and Aimless in CCP4 (CCP4, 1994). Parkin^S65N^ structure was determined by molecular replacement using Phaser in CCP4, WT-Parkin (pdb id: 5C1Z) was used as the search model. The initial model obtained from Phaser was further constructed and refined using Coot [75] and REFMAC5 in CCP4 [76], respectively. The final model of Parkin^S65N^ shows excellent geometry (electronic supplementary material, table S4).

### 4.17. Human genetic analysis

#### 4.17.1. Case 1 (S65N-M70)

Patient DNA samples were screened for variants using HaloPlex targeted sequencing of 82 selected PD-associated loci (Agilent Technologies, Santa Clara, CA, USA). DNA was extracted from peripheral blood by standard procedures. Sequencing was performed with a MiSeq sequencer (Illumina, San Diego, CA, USA), variant calling with Genome Analysis Toolkit [77] and the annotation with ANNOVAR [78]. The variants were confirmed by independent DNA sequencing method, Sanger sequencing. For the c.194G > A mutation in *PARK2* we used the following PCR and sequencing primers for PCR and sequencing: 5′-CTCGCATTTCATGTTTGACATTTCC; 5′-ATGCTCCATGCAGACTGCACTAAAG; sequencing 5′-CCTGCTGTCAGTGTGCAGAATG. The frequency of variants was screened from ExAC (http://exac.broadinstitute.org/) and gnomAD browsers (http://gnomad.broadinstitute.org) [79], the potential pathogenic effects were evaluated using CADD C-score [42] and conservation using UniProt [80].

#### 4.17.2. Case 2 (S65N-F60)

We downloaded the clinical data of case 2 (ID 3185) from the Parkinson's Progression Markers Initiative (PPMI) database (www.ppmi-info.org/data) on 13 August 2018. PPMI is a multi-centre, on-going longitudinal and observational study, which contains clinical, imaging and biological data from 454 PD patients and 215 control patients currently stored at the Laboratory of Neuro Imaging (LONI) database at University of California, Los Angles (UCLA). The cohort consists of 620 females and 828 males. The open-access repository is available online after registration at http://www.ppmi-info.org/. Informed consent was signed by the patients enrolled in the PPMI study. The patient's DNA whole-genome sequence data were downloaded from PPMI repository on 8–11 September 2018 and we filtered all coding variants in 82 PD-associated loci (used in-targeted sequencing panel of patient S65N-M70 (ID3185) excluding mitochondrial DNA) with a CADD C-score of over 20 and a carrier frequency less than 1%. Polyphen-2 [81] HVAR-based predictions are provided for each variant.

## Supplementary Material

Supplementary Data File

## Supplementary Material

Supplementary Figure 2

## Supplementary Material

Supplementary Figure 3

## Supplementary Material

Supplementary Figure 4

## Supplementary Material

Supplementary Figure 5

## Supplementary Material

Supplementary Figure 6

## Supplementary Material

Supplementary Figure 7

## Supplementary Material

Supplementary Figure 8

## Supplementary Material

Supplementary Figure 9

## Supplementary Material

Supplementary Table 1

## Supplementary Material

Supplementary Table 2

## Supplementary Material

Supplementary Table 3

## Supplementary Material

Supplementary Table 4

## Data accessibility

Coordinates and structure factors have been deposited with the PDB database (PDB ID 6HUE).
