# Supplementary material for: Phosphorylation of Parkin at serine 65 is essential for its activation in vivo
Source: Open Biol. 2018 Nov 7;8(11):180108. doi: 10.1098/rsob.180108 (PMC6282074; doi:10.1098/rsob.180108)
Supplement: Supplementary Table 1 [file rsob180108supp10.docx]

| Patient-ID | *S65N/S65N-M70* | *S65N/S65N-F60* |
| --- | --- | --- |
| Variant | Homozygous c.G194A (p.S65N) in PARK2, genomic location chr6:162683775 | |
| Sex | Male | Female |
| Age at onset | 38 | 53 |
| Age at diagnosis | 40 | 54 |
| Age at death | alive | alive |
| Disease duration (years) | 31 | 6 |
| Family history | - | - |
| Initial symptoms | rigidity of lower limbs, left side predominant, shortness of steps | bradykinesia and gait difficulty, right side |
| Motor Assessments | | |
| UPDRS (Total score and subscores) | 41/199 (I 0/18, II 9/52, III 27/108, IV 5/23) | NA |
| MDS-UPDRS I (1.1-1.6) | NA | 0* |
| MDS-UPDRS I: pq (1.7-1.13) | NA | 2* |
| MDS-UPDRS II: pq (2.1-2.13) | NA | 0* |
| MDS-UPDRS III (3.1-3.18, OFF) | NA | 20* |
| MDS-UPDRS III (3.1-3.18, ON) | NA | 3* |
| MDS-UPDRS IV (4.1-4.6) | NA | 0* |
| Hoehn & Yahr scale | 2.5 (Modified) | 2* |
| Resting tremor | - | slight/mild |
| Bradykinesia | + | + |
| Rigidity | + | + |
| Gait disturbances | + | + |
| Postural instability | + | slight |
| Assymetry at onset | + | + |
| Clinical response to levodopa | Good, current dosage 300-400mg/day | Not started (on Pramipexole, dosage 2.25mg/day) |
| Wearing off | + | infrequent |
| on/off | <25% off | no off-time/<25% |
| Levodopa induced dyskinesia | mild | NA |
| Hypomimia | mild | slight/mild |
| Dysarthria | mild to moderate | - |
| Hyperreflexia | hyporeflexia | (-)** |
| Non-motor/Other Symptoms | | |
| Orthostatic hypotension | - | - |
| Urinary urgency | - | slight |
| Constipation | - | - |
| Hallucination | - | - |
| Dementia | - | - |
| Depression | - | - |
| REM sleep behavior | - | - |
| DaT SCAN SBR Values*** | RC 1.71, LC 1.93, RP 1.19, LP 0.88 | RC 1.27, LC 1.28, RP 0.71, LP 0.71 |
| Other | multinodular goitre, osteoarthritis, varicose veins | acne, rash (neck), varicose veins, pterygium, bilateral inguinal hernia |
| pq: patient questionnaire; SBR: Striatal Binding Ratio, RC:right caudate, LC:left caudate, RP:right putamen, LP:left putamen, *latest assessment, **hyperreflexia observed on (only) one check-up, other times normal reflexes, ***different imaging facilities and protocols were used, results are not directly comparable (see Figure 5 for imaging of the patient S65N-M70 and www.ppmi-info.org. for the imaging of patient S65N-F60 by DaT scan method. | | |
