# Supplementary material for: Phosphorylation of Parkin at serine 65 is essential for its activation in vivo
Source: Open Biol. 2018 Nov 7;8(11):180108. doi: 10.1098/rsob.180108 (PMC6282074; doi:10.1098/rsob.180108)
Supplement: Supplementary Table 3 [file rsob180108supp12.docx]

**Supplementary Table 3**

| **Chr** | **Coordinate** | **R** | **A** | **Gene** | **Nucleotide change** | **AA Change** | **Zygosity** | **AF*** | **dbSNP** | **Polyphen2**** | **CADD ***** | **Comment** |
| --- | --- | --- | --- | --- | --- | --- | --- | --- | --- | --- | --- | --- |
| chr6 | 162683775 | C | T | PARK2 | exon3:c.G194A  (NM_004562) | p.S65N | hom | 8.179e-6 | rs754604402 | D | 25.5 | Novel |
| chr15 | 89873364 | C | G | POLG | exon3:c.G803C  (NM_001126131) | p.G268A | het | 0.0034 | rs61752784 | D | 26.6 | Linked to AR/sporadic PEO (1,2) |
| chr16 | 89986091 | G | A | MC1R | exon1:c.G425A  (NM_002386) | p.R142H | het | 0.0051 | rs11547464 | D | 28.6 | Likely not disease-linked |
| chr1 | 155205634 | T | C | GBA | exon10:c.A1226G  (NM_001005741) | p.N409S | het | 0.0022 | rs76763715 | B | 23.7 | Risk variant for late-onset PD (also named N370S) (3) |

Chr:chromosome, R:reference nucleotide, A:alternate nucleotide, AA:amino acid, het:heterozygous, hom:homozygous, AF:allele frequency, dbSNP:the Single Nucleotide Polymorphism Database reference, D:Probably damaging, B:Benign. *gnomAD total allele frequency, **Polyphen2 (HVAR-based) prediction, ***CADD-C Score v1.4 (PHRED)

**References**

1. Di Fonzo, Bordoni *et al*. (2003)
2. Graziewicz, Longley *et al*. (2006)
3. Sidransky, Nalls *et al*. (2009)
