# Supplementary material for: Phosphorylation of Parkin at serine 65 is essential for its activation in vivo
Source: Open Biol. 2018 Nov 7;8(11):180108. doi: 10.1098/rsob.180108 (PMC6282074; doi:10.1098/rsob.180108)
Supplement: Supplementary Table 4 [file rsob180108supp13.docx]

| **Parkin S65N** | |
| --- | --- |
| **Data collection** | |
| Wavelength | 0.966 |
| Resolution range | 47.98  - 2.85 (2.952  - 2.85) |
| Space group | P 21 21 21 |
| Cell dimensions |  |
| *a*, *b*, *c* (Å) | 66.4, 66.9, 206.7 |
| *α, β, γ* (°) | 90, 90, 90 |
| Total reflections | 60990 (8512) |
| Unique reflections | 21291 (3009) |
| Multiplicity | 2.9 (2.8) |
| Completeness (%) | 96.1 (94.7) |
| Mean I/sigma(I) | 4.5 (1.2) |
| Wilson B-factor | 56.97 |
| R-merge (%) | 12.5 (47.0) |
| CC1/2 | 0.96 (0.67) |
| **Refinement** |  |
| Reflections used in refinement | 20094 (1425) |
| Reflections used for R-free | 1095 (69) |
| R-work (%) | 22.3 (36.9) |
| R-free (%) | 25.5 (39.9) |
| Number of atoms |  |
| Protein | 6124 |
| Ligands/ion | 52 |
| Water | 159 |
| R.m.s. deviation |  |
| Bond lengths (Å) | 0.012 |
| Bond angles (°) | 1.58 |
| Ramachandran favored (%) | 93.0 |
| Ramachandran allowed (%) | 7.0 |
| **PDB ID** | 6HUE |

**Data collection and refinement statistics.**

**Statistics for the highest-resolution shell are shown in parentheses.**
